# Prognostic Value of the Lymph Node Ratio in Stage III Gastric Cancer Patients Undergoing Radical Resection

**DOI:** 10.1371/journal.pone.0096455

**Published:** 2014-05-08

**Authors:** Bin Ke, Xi-Na Song, Ning Liu, Ru-Peng Zhang, Chang-Li Wang, Han Liang

**Affiliations:** 1 Department of Gastric Cancer, Tianjin Medical University Cancer Institute and Hospital, National Clinical Research Center for Cancer, Key Laboratory of Cancer Prevention and Therapy, Tianjin, Ti-Yuan-Bei, He Xi District, Tianjin, People's Republic of China; 2 Department of Lung Cancer, Tianjin Medical University Cancer Institute and Hospital, Tianjin Lung Cancer Center, National Clinical Research Center for Cancer, Key Laboratory of Cancer Prevention and Therapy, Tianjin, Ti-Yuan-Bei, He Xi District, Tianjin, People's Republic of China; 3 Union Stemcell & Gene Engineering Co.,LTD, Nan Kai District, Tianjin, People's Republic of China; University at Buffalo, SUNY, United States of America

## Abstract

**Objective:**

The aim of this study was to investigate the prognostic value of metastatic lymph node ratio (LNR) in patients having radical resection for stage III gastric cancer.

**Methods:**

A total of 365 patients with stage III gastric cancer who underwent radical resection between 2002 and 2008 at Tianjin Medical University Cancer Institute and Hospital were analyzed. The cut-point survival analysis was adopted to determine the appropriate cutoffs for LNR. Kaplan–Meier survival curves and log-rank tests were used for the survival analysis.

**Results:**

By cut-point survival analysis, the LNR staging system was generated using 0.25 and 0.50 as the cutoff values. Pearson's correlation test revealed that the LNR was related with metastatic lymph nodes but not related with total harvested lymph nodes. Cox regression analysis showed that depth of invasion and LNR were the independent predictors of survival (p<0.05). There was a significant difference in survival between each pN stages classified by the LNR staging, however no significant difference was found in survival rate between each LNR stages classified by the pN staging.

**Conclusions:**

The LNR is an independent prognostic factor for survival in stage III gastric cancer and is superior to the pN category in TNM staging. It may be considered as a prognostic variable in future staging system.

## Introduction

Gastric cancer is one of the most common forms of cancer worldwide, with approximately one million new cases diagnosed each year [1]. More new cases of gastric cancer are diagnosed in China than in any other country around the world. Due to lack of effective screening in China, most gastric cancers are identified at an advanced stage [2], which is reflected by poor overall survival rates. Nowadays, the tumor-node-metastasis (TNM) system has become the principle method for assessing the prognosis of gastric cancer patients [3]. In 2010, the UICC published the 7^th^ edition TNM classification of malignant tumors for gastric cancer [4]. In this edition, the stage III was changed to contain three subdivisions: IIIa, IIIb, IIIc. The T4N+/TanyN3M0 classification which was defined as stage IV in the 6^th^ edition, was classified as the stage III according to the 7^th^ classification system. However, despite complete curative resection, the survival of patients with stage III gastric cancer is not satisfactory, even with standard, postoperative adjuvant chemotherapy. The 5-year survival rate of patients is less than 50% [5]. Therefore, it is important to identify prognostic factors for these patients and to better tailor treatment decisions.

Lymph node metastasis is one of the most important prognostic factors for gastric cancer [6]. The identified number of metastatic lymph nodes depends on the number of lymph nodes removed and examined. If the number of dissected and examined lymph nodes is small, down-migration of pN stage may occur, and conversely, if the number is large, upmigration of pN stage may occur, which is also referred to as stage migration in some references [7], [8]. The phenomenon of “stage migration” has been observed in 10% to 15% of cases [9], [10]. Thus, the current UICC TNM classification suggests that at least 16 lymph nodes should be examined for a precise assessment of pN stage [11]. Recently, the concept of the lymph node ratio (LNR), which is the proportion of metastatic to examined lymph nodes, has been proposed as a prognostic factor in gastric cancer patients [12] . This factor seems to obviate the problems of over-staging or under-staging inherent in the current TNM system [13]. A lot of studies suggested that the LNR was considered as a better prognostic factor for patients with gastric cancer than number of metastatic lymph nodes because it overcame the problem of pN staging bias [14]. However, the clinical significance of LNR is unclear in patients with stage III gastric cancer treated by radical surgery. Also, whether the LNR stage system of lymph node classification is a more accurate prognostic tool than the 7^th^ edition UICC pathological N (pN) stage in stage III gastric cancer patients is still unknown.

Therefore, the aim of this study was to assess the prognostic impact of the LNR for stage III gastric cancer, and to investigate whether the current pN staging system is appropriate for stage III gastric cancer and if LNR can be a predictor of survival.

## Patients and Methods

### Patients and treatment

From January 2002 to December 2008, a total of 365 patients with histologically confirmed primary gastric adenocarcinoma were diagnosed with stage III gastric cancer according to new 7^th^ edition TNM classification at Tianjin Medical University Cancer Institute and Hospital were initially screened for enrollment in this study. All cases underwent a total or subtotal gastrectomy with D2 lymphadenectomy, R0 resection, and the analysis of more than 15 lymph nodes. D2 lymphadenectomy was performed by experienced surgeons following the JRSGC guidelines [15]. All patients received postoperative adjuvant chemotherapy based on Platinum agents and Fluorouracil for 6 to 8 cycles.

The clinical and pathological data of the patients were prospectively collected and stored in a computer database. The database included the following information: name, gender, age, tumor location, tumor size, Borrmann classification, differentiation grade (undifferentiated and differentiated), TNM staging, type of surgery underwent, and follow-up conditions. All patients were followed up every 3 months for the first 2 years, every 6 months for the following 3 years and once a year thereafter. All the patients received at least 5 years follow-up or until death. For the purposes of this study, the last follow-up was performed on July 31, 2013.

### Ethics statement

The study was approved by the Research Ethics Committee of Tianjin Medical University Cancer Institute and Hospital, China. Written informed consents were obtained from all patients before participating in the study.

### Statistical methods

Pearson's correlation coefficient was used to study the relations between positive lymph nodes and retrieved lymph nodes, and LNR and retrieved lymph nodes, and LNR and positive lymph node count. The overall survival (OS) was calculated from the date of surgery to death or until the date of the last time the patient was known to be alive if the patient was not known to have died. The LNR was categorized by cutoffs determined using cut point survival analysis [16]. Survival curves and univariate analysis were calculated according to the Kaplan-Meier method, the log-rank test was used to evaluate statistically significant differences between two groups. Cox regression analysis was used in multivariate analysis of prognostic factors. Statistical significance was defined as *P*<0.05. Statistical analyses were performed using SPSS for Windows version 17.0 software (SPSS, Inc., Chicago, IL, USA).

## Results

### Clinicopathological parameters of patients

All of the patients with gastric cancer were in stage III in terms of the UICC TNM classification (7^th^ edition). The clinicopathological parameters of patients were summarized in Table 1. There were 249 males and 116 females at the age of 26–85 years (60.1±11.7). The tumor diameter was 6.1±2.8 cm. In total, 76 patients (20.8%) were in stage IIIA, 114 patients (31.2%) in stage IIIB, and 175 patients (48.0%) in stage IIIC. The median follow-up after surgery was 28.2 months (range, 1.5–96.7) for all patients. The median number of lymph nodes removed per patient was 22.0 (range, 16–72). The median number of positive lymph nodes was 7.0 (range, 1–67). The median LNR was 31.3% (range, 2.6%–100.0%).

**Table 1:** Clinical and pathologic characteristics of the patients.

Characteristics	No. of patients (%)
Gender	
Male	249(68.2)
Female	116(31.8)
Age	
≤60	183(50.1)
>60	182(49.9)
Size of tumor (cm)	
≤5	171(46.8)
>5	194(53.2)
Location of tumor	
Lower 1/3 stomach	128(35.1)
Middle 1/3 stomach	63(17.2)
Upper 1/3 stomach	101(27.7)
2/3 or more	73(20.0)
Histological type	
Differentiated	112(30.7)
Undifferentiated	253(69.3)
Borrmann classification	
I	38(10.4)
II	90(24.7)
III	150(41.1)
IV	87(23.8)
Depth of invasion	
pT_2–3_	23(6.3)
pT_4a_	259(71.0)
pT_4b_	83(22.7)
pN stage (UICC)	
pN_1_	65(17.8)
pN_2_	113(31.0)
pN_3a_	131(35.9)
pN_3b_	56(15.3)
LNR	
LNR1	158(43.3)
LNR2	95(26.0)
LNR3	112(30.7)
TNM stage	
IIIa	76(20.8)
IIIb	114(31.2)
IIIc	175(48.0)
Gastric resection	
Proximal gastrectomy	81(22.2)
Distal gastrectomy	155(42.5)
Total gastrectomy	129(35.3)

### Correlation between LNR and lymph node dissection

**Figure 1:**
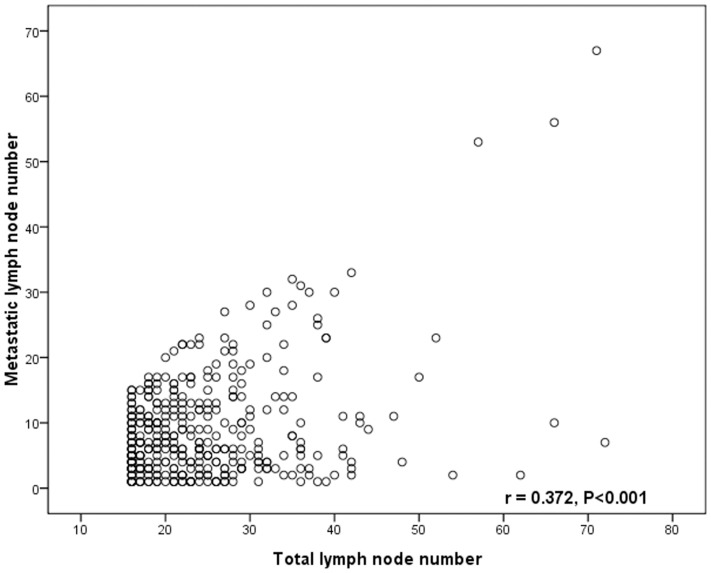
The correlation between the number of metastatic lymph nodes and the number of lymph node metastases. The metastatic lymph node number is significantly correlated with total number of lymph node harvested. (r = 0.372, *p*<0.001)

**Figure 2:**
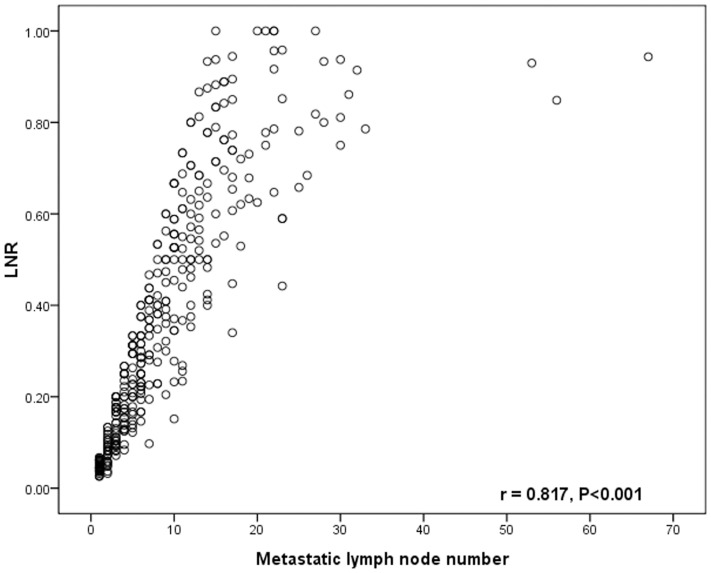
The correlation between the LNR and the number of lymph node metastases. The LNR is closely correlated with the number of lymph node metastases. (r = 0.817, *p*<0.001)

**Figure 3:**
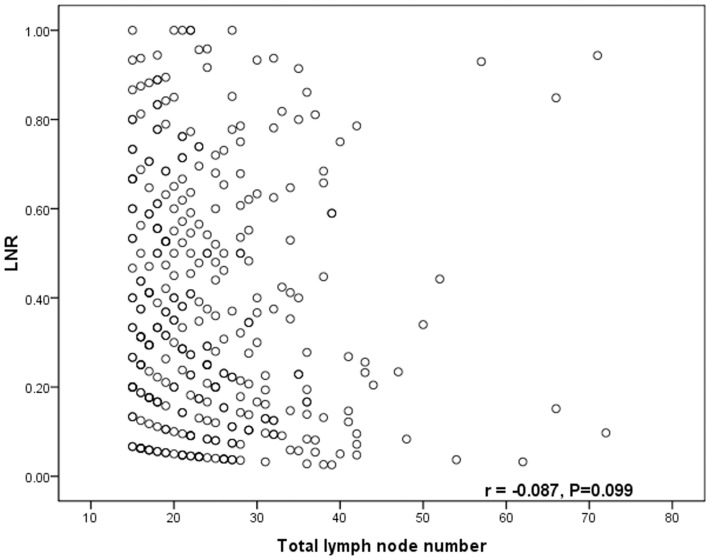
The correlation between the LNR and the number of retrieved lymph nodes. The LNR is not related with the total number of lymph node harvested. (r = 0.817, *p*<0.001)

Pearson's correlation test showed that the number of metastatic lymph nodes was closely correlated with the number of total harvested lymph nodes (*r* = 0.372, *P*<0.001, Figure 1), the LNR was significantly related with the number of metastatic lymph nodes (*r* = 0.817, *P*<0.001, Figure 2), but the LNR was not related with the number of total harvested lymph nodes (*r* = −0.087, *P* = 0.099, Figure 3).

### Examine the classification of LNR

**Figure 4:**
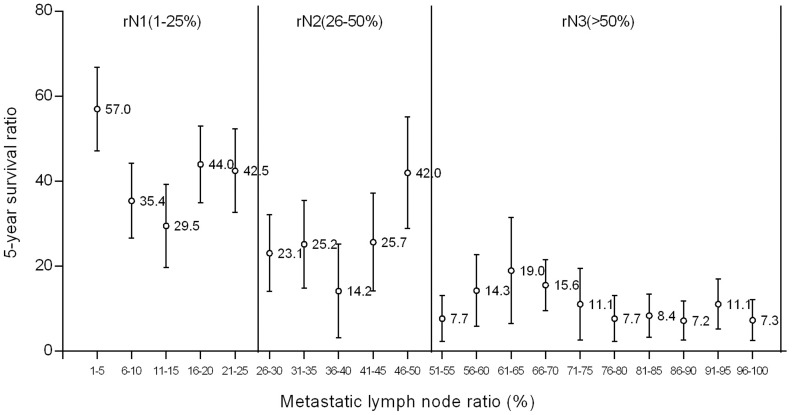
Observed 5-year survival rates depending on the LNR. (Error bar shows 95% confidence intervals)

To detection the best cutoff of LNR, we estimated the 5-year survival rates (5-YSR) with 95% confidence intervals in relationship to metastatic lymph node ratio at interval of 5%. Figure 3 showed that the appropriate cutoff of LNR for verification of statistically significant survival differences between resulting subgroup were 25% and 50% (Figure 4). We separated the population into three subgroups with remarkably different survival rates. The cutoff values of the LNR were set at LNR1 (0.01–0.25), LNR2 (0.26–0.50), and LNR3 (>0.50). It was calculated that 158 patients (43.3%) were restaged in LNR1, 95 patients (26.0%) were restaged in LNR2 and 112 patients (30.7%) were restaged in LNR3 according to the metastatic lymph nodes ratio classification.

### Prognosis analysis according to different N staging

**Figure 5:**
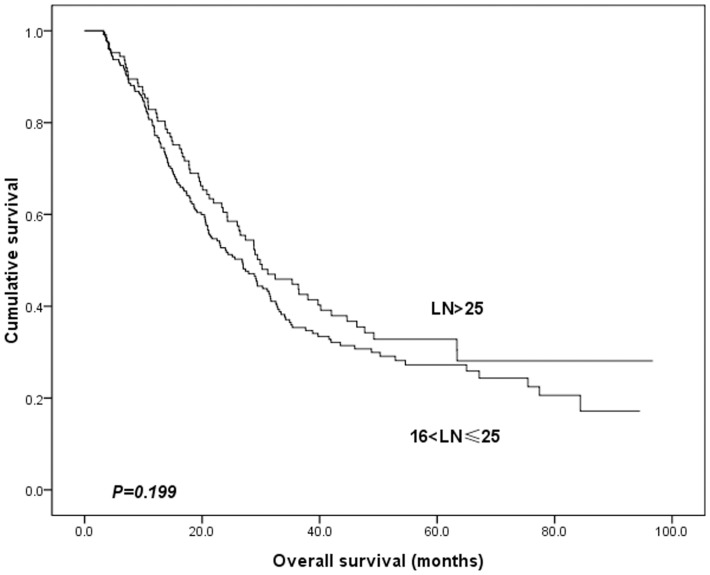
Survival curve for stage III gastric cancer patients according to the number of lymph nodes examined. (p = 0.199)

We also investigated the impact of the number of lymph nodes retrieved on OS rates according to different N staging systems. There were 239 and 126 patients who had 16–25 and more than 25 lymph nodes harvested, respectively. Their median survival times were 26.8 and 29.9 months, respectively (p = 0.199). The results are shown in figure 5. As shown in table 2, we stratified patients into two groups according to the number of lymph nodes examined (less or greater than 25). Differences between the survival rates of those two groups of patients was not statistically significant in both the pN and LNR staging system.

**Table 2:** Overall survival rates base on pN and LNR classification according to the number of lymph nodes examined.

N stage	16<LN≤25		LN>25			P value
	Case	5-year survival (%)	Case	5-year survival (%)		
pN1	45	36.9	20	53.0	0.148	
pN2	74	35.1	39	40.8	0.495	
pN3	120	19.3	67	17.4	0.552	
LNR1	90	42.9	68	45.5	0.564	
LNR2	70	25.2	25	26.7	0.822	
LNR3	79	10.7	33	12.5	0.594	

### Univariate and multivariate survival analysis

**Figure 6:**
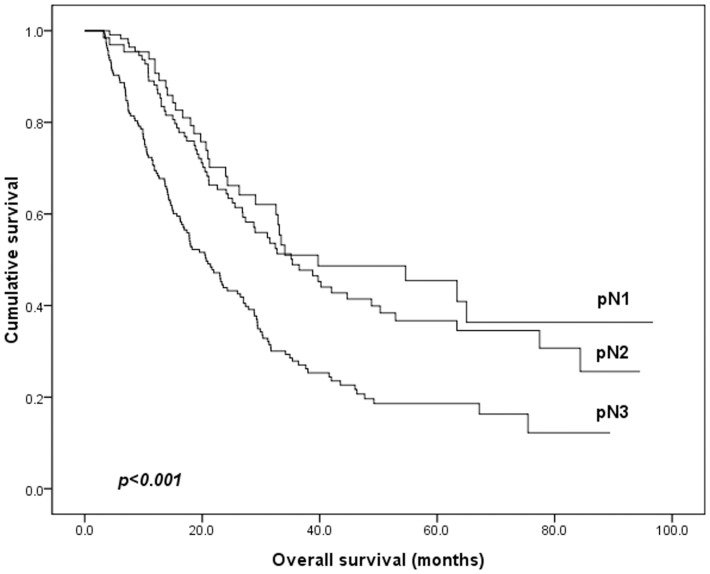
Survival curve for stage III gastric cancer patients according to 7^th^ UICC TNM classification pN staging. (p<0.001)

**Figure 7:**
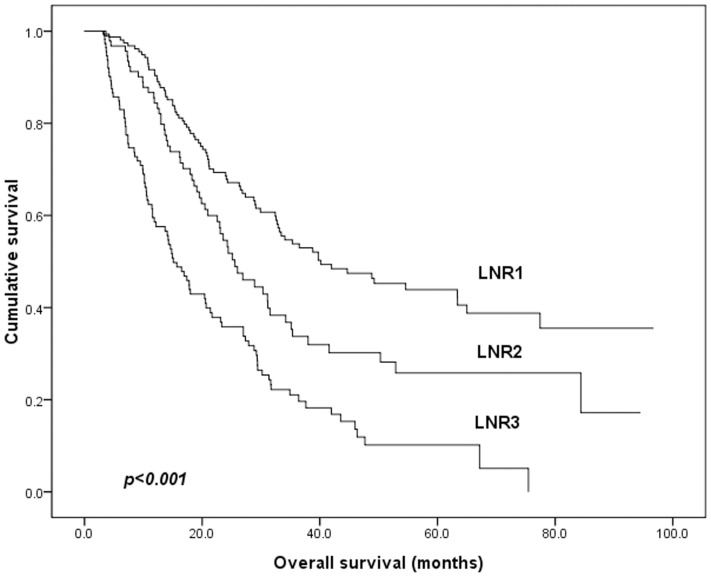
Survival curve for stage III gastric cancer patients based on the LNR staging. (p<0.001)

The median survival time for all 365 patients was 27.5 months and 5-YSR OS was 29.7%. The 5-YSR of pN1, pN2 and pN3 patients were 43.2%, 35.6% and 22.3%, respectively (Figure 6); The 5-YSR of LNR1, LNR2 and LNR3 patients were 43.9%, 25.8% and 10.2%, respectively (Figure 7). The clinicopathological variables tested in univariate analysis are shown in table 3. We found that age, the size of tumor, T staging, pN staging, and LNR staging (all p<0.05) had statistically significant associations with OS of stage III gastric cancer patients after curative surgery, while sex, histological type, Borrmann classification and type of gastrectomy were not influential to OS (p>0.05). All of these potential important factors identified in univariate analysis were included in a multivariate Cox proportional hazard model for analysis, (Table 4). Depth of tumor invasion and LNR were identified as the significantly independent prognostic factors for OS of all patients.

**Table 3:** Univariate analysis of factors affecting OS of 365 gastric cancer patients after curative surgery.

Characteristics	Case	5-year survival (%)	χ^2^	*P*-value
Gender			0.130	0.719
Male	249	27.5		
Female	116	31.4		
Age			6.857	0.009
≤60	183	35.0		
>60	182	20.6		
Size of tumor (cm)			4.320	0.038
≤5	171	35.1		
>5	194	23.6		
Location of tumor			0.890	0.828
Lower 1/3 stomach	128	32.6		
Middle 1/3 stomach	63	24.9		
Upper 1/3 stomach	101	26.2		
2/3 or more	73	23.1		
Histological type			2.166	0.141
Differentiated	112	33.8		
Undifferentiated	253	27.8		
Borrmann classification			2.209	0.530
I	38	38.2		
II	90	32.5		
III	150	28.6		
IV	87	22.9		
Depth of invasion			12.458	0.002
pT2-3	22	44.2		
pT4a	260	31.4		
pT4b	83	17.8		
pN stage (UICC)			26.413	0.000
pN1	65	43.2		
pN2	113	35.6		
pN3	187	18.6		
LNR			53.096	0.000
LNR1	158	43.9		
LNR2	95	25.8		
LNR3	112	10.2		
Type of gastrectomy			0.857	0.651
Proximal gastrectomy	81	29.1		
Distal gastrectomy	155	31.4		
Total gastrectomy	129	22.0		

**Table 4:** Multivariate analysis of factors affecting OS of Stage III gastric cancer patients.

Parameter	Significance	HR	95% CI	
			Lower	Upper
Age	0.755	1.054	0.756	1.471
Size of tumor	0.427	1.119	0.848	1.477
Depth of invasion	0.001	1.594	1.202	2.115
pN stage	0.730	1.044	0.819	1.330
LNR	0.000	1.636	1.255	2.132

(HR Hazrd ratio; CI, confidence interval)

### Correlation analysis between pN classification and LNR classification

In our study, we examined 5-year survival rates on the basis of pN classification according to the LNR staging system. As shown in Table 5, for patients in each LNR classification, prognosis was highly homologous between those in different pN classifications. However, for patients in each pN classifications, significant differences in survival could always be observed among patients in different LNR classifications. This find indicated that the LNR classification is more appropriate than pN classification for categorization of metastatic lymph nodes for evaluating the OS of Stage III gastric cancer patients after curative surgery.

**Table 5:** 5-year survival rates (5-YSR) on the basis of pN stage according to the LNR stages.

pN staging	LNR1		LNR2		LNR3		χ^2^	*P* ^a^ value
	No	5-YSR(%)	No	5-YSR(%)	No	5-YSR(%)		
pN1	65	43.2					-	-
pN2	84	41.8	29	16.2			5.467	**0.019**
pN3	9	33.3	66	31.8	112	10.2	15.585	**0.000**
X^2^		0.015		0.606		-		
*P* ^b^ value		0.993		0.436		-		

*P* ^a^ value: Comparison of survival rates between different LNR groups.

*P* ^b^ value: Comparison of survival rates between different pN groups.

## Discussion

Amongst prognostic factors predicting improved survival, lymph node metastasis is well known to be one of the most important prognostic factors in gastric cancer. Accurate pN staging is of great importance for determining prognosis and therapy of gastric cancer patients. The TNM staging system has become accepted worldwide as the most important reference for treatment planning in clinical oncology and prognosis prediction for gastric cancer [3]. In UICC/AJCC system, the nodal status is classified according to the number of metastatic lymph nodes as pN0, no lymph node metastasis; pN1, 1-2 metastatic lymph nodes; pN2, 3–6 metastatic lymph nodes; pN3a, 7–15 metastatic lymph nodes; pN3b, >15 metastatic lymph nodes [4]. In order to avoid stage migration, the UICC/AJCC classification recommended that 15 or more lymph nodes should be evaluated for the accurate staging of gastric cancer [11]. However, recent evidence also indicates that the number of total lymph nodes harvested is an independent prognostic factor for gastric cancer outcome [17], [18]. To resolve these limitations, several studies have suggested that the LNR, which is defined as the ratio of the number of metastatic lymph nodes to the total number of nodes harvested, may be a more reliable and accurate prognostic indicator than the number of metastatic lymph nodes alone [11]–[14]. In this study, we classified patients according into LNR quartiles and determined cutoff levels that differentiated between oncologic results. Moreover, we investigated the prognostic value of the LNR staging system in the stage III gastric cancer patients who underwent curative resection. To minimize the stage migration of pN, all patients enrolled in this study should undergo histopathologic examination of more than 15 lymph nodes (mean 22.0, range, 16–72).

According to Pearson's correlation test of all the patients, the LNR significantly increased with the number of metastatic lymph nodes, while no correlation exists between the LNR and the total number of harvested lymph nodes. This study is in agreement with the data from Huang et al [19].This finding indicated that the more the number of metastatic lymph node is, the higher the LNR will be, indicating that gastric cancer patients with higher LNR have a greater risk of death after surgery and a shorter survival time, and LNR had an oncologic impact as the number of metastatic lymph nodes. Furthermore, the results showed that LNR can be used as a potent predictor of survival for stage III gastric cancer patients regardless the total number of lymph nodes harvested.

However, the best cut-off of LNR staging was still in debate. Several levels of cut-off value have been reported in previous studies [9]–[14], [19]–[20], which might reflect differences in patient number, the proportion of early to advanced stage, the degree of lymph node dissection, or the differences in the histopathological methods of lymph node examination. All of these studies confirm the LNR as a significant prognostic factor. In this study, in order to find out the optimal cut-off value of LNR, we initially categorized the patients by every 0.05 interval of LNR, LNR was determined by the best cutoff approach in terms of the long-rank test. The best cutoff points were 0.25 and 0.50, similar to Inoue's study [20]. And the patients in our study are divided into three groups of 0<LNR≤0.25, 0.25<LNR≤0.50 and LNR>0.50, the difference of OS between these three groups were more significant. However, the LNR was higher in patients with stage III gastric cancer than in those with stage I or II disease. Whether this LNR classification is appropriate for all stage gastric cancer patients still need be evaluated further in a large prospective randomized clinical trial. Thus, the effort to find the optimal cutoff is necessary to popularize the global usage of the LNR system.

In univariate analysis, age, the size of tumor, the depth of tumor invasion, pN staging, and LNR staging were found to have statistically significant association with OS of stage III gastric cancer patients. However, in a multivariate Cox Hazard model, only the depth of tumor invasion (*p* = 0.001) and (LNR *p* = 0.000) were the important independent prognostic factors for OS evalutaion but not the number of metastatic lymph nodes (pN) (*p* = 0.730). In this study, the LNR was proved to be one of the most significant prognostic indicators in stage III gastric cancer patients. Our finding is consistent with many studies that the LNR provided a better classification of patients' prognostic risk profile than the pN classification system.

As described in previous reports, more than 15 lymph nodes are required for the adoption of the current UICC/TNM pN staging system [11]. However, Kong et al [9] performed a retrospective survival analysis of 8,949 gastric cancer patients who underwent curative surgery, the results indicated that because the proportion of advanced pN stage cases substantially increases as the number of lymph nodes increases, the minimum number of 15 lymph nodes required for “accurate staging” was previously suggested cannot be enough for accurate staging. We found that the prognosis of patients with 16–25 harvested lymph nodes was not significantly different from those with more than 25 lymph nodes harvested. We believe that the pN and LNR staging system would be accurate when more than 15 lymph nodes examined. Our founding is similar to the AJCC's recommendation that a minimum of 15 lymph nodes should be harvested for adequate staging. Our study result indicated that the number of metastatic lymph nodes identified may be influenced by the total number of lymph nodes examined, thus increasing the probability of stage migration. However, the number of lymph nodes identified depends on the surgical and pathologic procedures, and there might were more lymph nodes metastasis in stage III gastric cancer patients than stage I or II. Thus, the patients underwent standard surgery and the pathologists make effort to find all possible lymph nodes are necessary for accurate pN staging. These results imply limitations of the existing UICC/TNM pN staging system, which depends on only the number of metastatic lymph nodes, and suggest the need for a prognostic classification system based on LNR, reflecting the numbers of collected lymph nodes.

Many studies have shown that the clinical significance of the LNR was not related with the number of the harvested lymph nodes in gastric cancer [11], [21], but there were still others which showed contrary results [22], [23]. According to a study of Xu et al. [21], the LNR is an independent prognostic factor after D2 resection, regardless of the number of lymph nodes examined, and in study of Chen et al. [11], for those patients who with a small number of harvested lymph nodes, the LNR was able to predict survival better than TNM pN staging system. In this study, all patients with a number of collected lymph nodes of more than 15, and Pearson's correlations test showed that the LNR was not related with the number of total harvested lymph nodes, and this finding was similar with Xu's study [21]. Furthermore, in the present study, we compared the survival rates for patients classified by LNR category or by pN category, we found that a significant difference was observed in survival rates between lymph-node stages classified by the LNR category, but no significant difference was found in survival rates between each LNR stages classified by the pN stages. This implies that patients with identical LNR, even with a different number of detected metastatic nodes, will have a similar outcome. Conversely, among patients with the same number of metastatic nodes, those with a higher LNR will have an unfavorable outcome. This indicated that the UICC/TNM pN classification can demonstrate stage migration and heterogeneous stratification for disease-specific survival, and the LNR classification had the better prognostic homogeneity than pN classification. Ueno et al [24] recommended that the homogeneity within subgroups (small differences in survival among patients with the same stage) was a significant feature of a better staging system. Thus, we think the LNR classification is superior to the pN classifications and it can contribute to accuracy in prognostic assessment.

Although the data were collected prospectively, this study has limitations associated with the retrospective nature of its design. This may have introduced a selection bias. Another limitation was that the patients who did not receive chemotherapy were excluded from this study. Even with these limitations, however, we believe our sampling design to be adequate for assessing the usefulness of ratio-based LNR staging.

In conclusion, our result demonstrated that LNR is superior to N-stage in predicting stage III gastric cancer patients' outcome, and classification according to LNR can avoid the stage migration phenomenon related to the UICC/TNM staging system, especially when a radical resection has been performed and sufficient lymph nodes was retrieved. We suggest that a new algorithm incorporating the LNR can be added to the 7th UICC/TNM staging system. Furthermore, a LNR value of 25%, 50% could be an ideal cutoff point. However, the small sample size limited the conclusion of our study, in order to clarify the real prognostic value of the LNR and to identify best cutoff value of LNR, further multiple-center studies with a large population are required.
